# Hepathic, biochemical, hematological, and histological effects of the ultracavitation in rabbits livers ^1^

**DOI:** 10.1590/s0102-865020200040000003

**Published:** 2020-06-19

**Authors:** Patrícia Froes Meyer, Janiele Ferreira da Silva Sousa, Rejane Vilar da Rocha, José Queiroz, Oscar Ariel Ronzio, Rodrigo Marcel Valentim da Silva, Ana Camila de Medeiros Manso, Afra Rafaelli Magalhães de Almeida, Camila Procopio Andrada

**Affiliations:** I PhD in Health Sciences, and Assistant Professor, Centro Universitário do Rio Grande do Norte (UNIRN), Natal-RN, Brazil. Scientific and intellectual content of the study.; II Graduate student in Physiotherapist, UnP, Natal-RN, Brazil. Acquisition of data, manuscript preparation.; III PhD in Health Sciences, Universidade Federal do Rio Grande do Norte (UFRN), Natal-RN, Brazil. Biochemical analysis.; IV PhD in Health Sciences, Universidad Maimnides (Umai), Ciudad Autonoma de Buenos Aires, and Universidad Nacional Arturo Jauretche F. Varela, Argentina. Design of the study, critical revision.; Universidad Nacional Arturo Jauretche F. Varela, Argentina; V Fellow PhD degree in Physiotherapy, UFRN, and Assistant Professor, Physical Therapy Course, Faculdade Estácio de Natal, Brazil. Statistical analysis, manuscript preparation.; VI Specialist, Specialization in Functional Dermato Physical Therapy UnP, Natal-RN, Brazil. Acquisition of data, manuscript preparation.; VII Graduate student, Faculty of Medicine, UnP, Natal-RN, Brazil. Manuscript preparation.

**Keywords:** Lipodystrophy, Metabolism, Adipose Tissue, Rabbits

## Abstract

**Purpose:**

To collect data capable of pointing out the effects of the ultracavitation treatment on the liver of rabbits after adipose tissue application, by means of histological analyses of the liver and hematological and biochemical exams.

**Methods:**

This is an experimental study with 12 albino rabbits as sample, which were divided into 3 groups and submitted to a hypercaloric diet for one month. Subsequently, subjects underwent UCV treatment: 3 minutes, 30 W, continuous mode at 100%, every 2 ERAS = 441.02 J/cm^2^, intensity of 10w/cm^2^. They were then euthanized and underwent biopsy after 24 hours.

**Results:**

After 48 hours from the ultracavitation treatment, the animals’ livers presented greater amount of fat infiltration if compared to the amount presented 96 hours after the treatment. However, laboratory tests showed no alterations. Values were maintained within normal parameters of cholesterol, triglycerides, liver enzymes, hemoglobin and hematocrit levels.

**Conclusions:**

This study has identified that infiltrates may appear on livers after the treatment, despite high hematological and biochemical tests results. The fat infiltrates reduction 96 h after treatment suggests lower risks to animal health, if the period between applications is respected.

## Introduction

The high-intensity ultrasound device, also named *ultracavitator* , stands out among the non-invasive techniques that aim to safely and effectively meet the growing demand for methods of localized lipodystrophy reduction, even replacing previously utilized aggressive and invasive methods of adipose tissue reduction. This equipment is used in procedures of therapeutic purpose due to its efficient reduction of the adipose panicle, being recently reformulated and introduced in the segment of esthetic medicine^1 , 2^ .

The tissue response to high-intensity ultrasound treatment is the increase in the number of macrophages that surround and transport lipids and cellular debris away from the treated area. Most of the treated adipose tissue is reabsorbed within 8 to 12 weeks after application, with up to 95% absorbance after 18 weeks. The esthetic result is an overall reduction of the local adipose tissue volume, which promotes great patient satisfaction and, in addition, does not present any relevant changes in the dosages of basal free fatty acids, nor any changes in total cholesterol and triglyceride levels after the procedure^3^ .

In contrast, Ronzio *et al.* ^4^ report differences in the cholesterol data of one patient receiving ultracavitation in the anterolateral thigh region during 4 consecutive weekly sessions, with a 10-minute application in an area with twice the effective radiation (ERA), in full cycle at 50 W, 40 kHZ.

The purpose of this study was to collect data capable of identifying the effects of ultracavitation (UCV) on the liver of rabbits after the application to their adipose tissue, through histological analysis of the liver and hematological and biochemical examinations, aiming to clarify if the UCV application brings risks to health.

## Methods

It is an experimental study which was carried out in the laboratory of animal experimentation, in the Universidade Potiguar (UnP) laboratory.

The cohort consisted of 12 rabbits ( *Oryctolaguscuniculus* species, *Leporidae* family, albino type) of both genders, of between 2.7 kg and 500 g-body weight, measured with a precision scale (Balmak ELP® 06/15/30). The animals were allocated at the *vivarium* of UnP, and fed a hypercaloric diet for three months, and water was offered for rapid weight gain. The cohort was randomly and equally divided into 3 groups as shown in Table 1 . The first group (G1) was analyzed 48 h after treatment. The second group (G2) was the control group, with two rabbits being analyzed 48 and the other two analyzed 96 h after treatment. The third group (G3) was analyzed 96 hours after treatment.

**Table 1:** Procedures performed in each group.

	Number of animals	Examination and euthanasia	Treatment
Group 1	4 animals	48h	UCV
Group 2	2 animals 2 animals	48hs 96hs	Control without treatment
Group 3	4 animals	96hs	UCV

The animals were initially submitted to anesthesia using the dissociative anesthetic (Zoletil^TM^ 50) intramuscularly applied to the left quadriceps. The anesthetic dosage was calculated according to animal weight at 15.0 to 30.0 mg/kg. Then, abdominal hair trimming was manually performed with scissors.

G1 and G3 sample animals underwent UCV treatment with the device model Liposonic, brand Meditea (Argentina). The 3-minutes protocol was carried out based on the following parameters: 30 W, continuous mode at 100%, every 2 ERAS = 441.02 J/cm^2^ , intensity of 10 W/cm^2^ . The local asepsis was performed with sterile gloves, sterile gauze and 2% Chlorhexidine Digluconate, aiming to perform a cardiac puncture for blood collection in all groups, and with the proper care to avoid cross-infection caused by external agents. Aspartate aminotransferase (AST), alanine aminotransferase (ALT), cholesterol, triglycerides, hematocrits, and hemoglobin were analyzed.

The animals were euthanized in a gas chamber after the UCV procedure, in preparation for liver removal. The subjects were positioned in dorsal decubitus with all four legs restrained and the liver was surgically removed and fixed in formaldehyde solution at 10% for 24 h before being forwarded to histology laboratory for analyses. A biopsy of the skin and liver fragments was performed 48 and 96 h after UCV application, respectively, in the samples from G1 and G3 groups.

**Figure 1:**
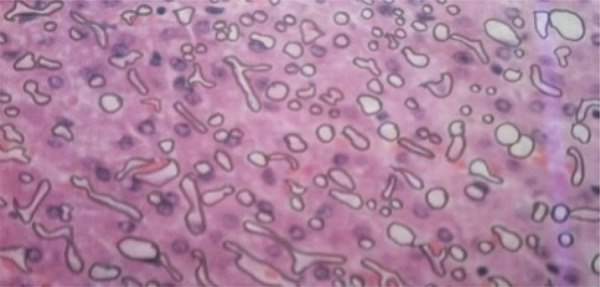
Photo of the hepatic tissue with contoured areas of fat infiltrates.

All the liver fragments were stored in individual containers with formaldehyde, with their proper specifications, and sent to a professional pathologist for histological analysis. Once the histological photos were taken, they were submitted to the AutoCAD program, version 2016 (English), in which the counting was done after the areas where fat infiltrations could be identified were marked/outlined, as shown in Figure 1 .

Two photos from each group were randomly selected for optical microscopy analysis (x400 magnification.)

The animal blood samples were then submitted to laboratory, hematological, and biochemical tests. An evaluation of the adipose cells number in the liver of the animals through the histological photos by the AutoCAD program was also carried out and, histological alterations were observed as well and described by three pathologists blindly. Quantitative data from the blood test and the AutoCAD program were presented in the form of tables and the findings were described.

For statistical analysis, the two-way ANOVA test was used to compare the analyzed groups.

## Results

**Figure 2:**
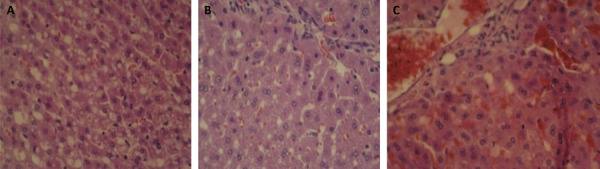
A. Photomicrography of liver tissue submitted to UCV after treatment - G1 after 48h. B. Photomicrography of liver tissue submitted to UCV after treatment - G3 - 96h. C. Photomicrography of untreated hepatic tissue - G2 - 48h and 96h (HE x400).

The histological analysis showed increasing amount of fat infiltrates compared to Figure 2B (group G2). However, G1 presented less fat infiltrates.

An increase in fat infiltrates in the treated groups G1 (48h) and G3 (96h) was detected, when compared to the control group (G2).

Table 2 displays the infiltration count data completed with the assistance of AutoCAD program application on two random histological photos, and the data present the average of fat infiltrates found in the animals’ livers.

**Table 2:** Mean number of infiltrates found in histological photos of each group.

Number of fat infiltrates in the liver	G1	G2	G3
Photo 1	95±4.5	35±2.0	78±7.1
Photo 2	241±14.5	35±2.1	145±9.8

The biochemical and hematological material was collected through cardiac puncture post UCV. After analysis, it was observed that the values remained within normal range. Data of discrete alteration was selected: biochemicals (cholesterol, triglycerides, AST, and ALT), and hematological (hematocrits and hemoglobin), and Table 3 compares the values among the three groups.

**Table 3:** Results of comparison means and standard deviation among groups - cholesterol, triglycerides, AST, ALT, hematocrits, and hemoglobin values.

Group	Cholesterol	Triglycerides	AST	ALT	Hematocrits	Hemoglobin
G1 (Treated)	66.25±4.5	43.3±7.1	36.25±12.9	57.5±11.4	46.16±15;1	12.6±6.2
G2 (Control)	64.66±5.9	53±9.3	58.25±13.1	55.75±9.2	45.4±8.2	12.3±5.4
G3 (Treated)	52.75±6.1	40.5±8.2	21±10.9	36.5±8.9	40.3±7.1	11.4±3.8

Regarding the biochemical (cholesterol, triglycerides, AST, and ALT) and hematological results (hematocrit and hemoglobin) submitted to statistical analysis; no significant results were found among the values when comparing groups that underwent treatment and the control group. From the data in Table 4 , no relevant results were observed, which may be attributed to possible alterations in cholesterol, triglycerides, AST, ALT, hematocrit, and hemoglobin, compared to reference values for these animals^5^ .

**Table 4:** Comparation Mean biochemical and hematological values - ANOVA.

		F	P valor
Cholesterol	Among Groups	0.19	0.83
Triglycerides	Among Groups	1.64	0.25
ALT	Among Groups	1.13	0.36
AST	Among Groups	0.712	0.51
Hematocrit	Among Groups	1.800	0.22
Hemogram	Among Groups	0.006	0.99

The two-way ANOVA test was applied, with a 95% significance level.

## Discussion

A possible explanation for the results of the histological analysis to demonstrate the presence of fat infiltrates in the animals’ livers is that they were probably a consequence of the weight gain promoted by the hypercaloric diet the subjects underwent until treatment. However, in a comparative analysis, the treated groups (G1 and G3) presented greater amounts of fat infiltrates in the hepatic tissue. Such findings may indicate that the high intensity and high frequency protocol of UCV applied in this study was able to promote the increase of fat presence in the hepatic parenchyma, being its highest incidence 48 h after treatment, which reinforces the affirmations of a few studies^1 - 4^ . Despite being caged and not performing any physical activity, in 96 h after treatment, reduction in the amount of fat was identified.

Zucco *et al.* ^6^ corroborate with such finding by reporting that there is extravasation of the adipose cell content, known as triglycerides, when submitted to cavitation, causing the adipocyte cells membranes to rupture. For the inner content to be eliminated, lipase activation takes place, connecting glycerol and free fatty acids which, after being oxidized in the tissues, use energy, and are released into the interstitial fluid and naturally delivered via vascular and lymphatic system to the liver, where metabolization occurs without distinction between the fat originated from destroyed fat cells and the fat derived from food consumption. The metabolization of the adipocyte occurs physiologically in the organism.

According to Spencer, after the application of the ultracavitor, fat clearance is done by physiological pathways such as the lymphatic, venous, and immunological systems. Triglycerides from cells that have undergone lipolysis are released into the interstitial fluid where they are gradually transported through the lymphatic or venous system to the liver and are used by metabolic pathways in a process that may last several hours or several days. Cellular debris are allowed by the normal inflammatory response for phagocytosis. Such degradation products are safely transferred through the blood. Moreno *et al.* ^5^ reported that triglyceride levels may rise within normal levels in the first 48 h after application of the focused ultrasound device, demonstrating that the equipment operates under a safe technique procedure^7 , 8^ .

The description of the fat increase process in the hepatic parenchyma after UCV exists in literature, superficially and without much clarification as to the time and consequences to patient’s health. In addition, there is disagreement among authors about what happens and about the safety in the use of UCV devices. It should be considered that the study by Moreno *et al.* ^5^ used a different device (focused ultrasound) with different parameters from the UCV device used in this study.

Studies by Moreno *et al.* ^5^ report that, histologically, the presence of adipocyte cell lysis surrounded by vessels and intact nerves was confirmed. After fat cells rupture, their content, mostly composed of triglycerides, is dispersed in the interstitial space, where it is slowly metabolized by the endogenous lipase into fatty acids and glycerol, and the fatty acids are transported by the lymphatic system to the liver, where it is processed, similarly to dietary fatty acids^1 , 8^ .

Although it is stated in literature that in a normal situation, according to Turaça, there is a relationship between physical exercises and the improvement of the lipid profile, meaning, decrease in triglycerides, VLDL, LDL and increase in HDL, and that more intense exercises with greater energy expenditure bring greater benefit comparing to moderate intensity and low-energy expenditure exercises, studies have not yet correlated this with the application of UCV devices and the importance of physical exercise concomitant with the treatment program. Such recommendation is still empirical, since the liver is the central manager of lipid metabolism, controlling the distribution of fats by the body and its use^9^ .

A study by Brito *et al* .^7^ , with a population of 22 female volunteers, aged 20 to 39 years, amongst which some did and some did not practice physical activities, showed a significant reduction in the lipid profile in the values of total cholesterol and LDL in the group of individuals who practiced physical activities after the use of the ultrasound cavitator in abdominal adipose tissue. It is worth mentioning that repeated applications with the UCV device in a period inferior to 96 hours, according to this research, could bring risk of hepatic steatosis if the patient did not undergo excessive metabolic expenditure. Nevertheless, more studies for the clarification of this process are required, including the ones submitting treated animals to an aerobic activity to measure the possible protective effect of this exercise^10^ .

There are still controversies regarding the presence of fatty infiltrates in the liver and the connection with the UCV treatment. According to Niwa *et al* .^8^ , and Fatemi *et al* .^12^ , cavitational ultrasound is a non-invasive, effective, and safe localized fat treatment.

The technique delivers energy to the skin surface at a relatively low intensity, but with strong focus on subcutaneous fat. On the skin surface, the ultrasonic intensity energy is low enough not to cause harm or damage. There is no risk of lymphatic, venous or hepatic overload, as the body’s ability to excrete cavitated fat is much greater than the amount of fat released by the cavitational ultrasound procedure^11 , 12^ .

Regarding the laboratory exams analyses (hematological and biochemical), no relevant changes were found. Jewell *et al.* ^13^ . also stated in a study performed with 26 swine samples, that there were no changes in lipid panels or in liver function tests after treatment using high intensity focused ultrasound (HIFU) on the ventral (abdominal) surface of the samples^10^ . In contrast, Ronzio *et al* .^4^ stated that there was difference in the cholesterol data when the UCV treatment was performed in a patient who did not present previous alterations in her exams.

One limitation in this study is its application in animals and the correlation with humans. Further research is essential to analyze, for example, the effects of physical activities after UCV treatment, and studies that analyze human liver tissue, to better confirm the results presented in this research.

## Conclusions

The effects of the UCV treatment on rabbits’ livers showed an increase in fat infiltrates in the hepatic tissue 48h after treatment, evidenced in the quantitative analysis and in the histological descriptive analysis. However, there were no evident changes in the laboratory tests results, which leads to the conclusion that infiltrates may appear after treatment without the increase in the hematological and biochemical exams. A decrease in the quantity of fat infiltrates was observed 96h after treatment, suggesting lower risk to animal health if this range of applications is respected.

## Footnotes

1 Research performed at Department of Physiotherapy, Universidade Potiguar (UnP), Natal-RN, Brazil.
